# Extended Codebook with Multispectral Sequences for Background Subtraction †

**DOI:** 10.3390/s19030703

**Published:** 2019-02-08

**Authors:** Rongrong Liu, Yassine Ruichek, Mohammed El Bagdouri

**Affiliations:** Connaissance et Intelligence Artificielle Distribuées (CIAD), University Bourgogne Franche-Comté, UTBM, F-90010 Belfort, France; yassine.ruichek@utbm.fr (Y.R.); mohammed.el-bagdouri@utbm.fr (M.E.B.)

**Keywords:** background subtraction, multispectral sequences, codebook, self-adaptive, spectral information divergence

## Abstract

The Codebook model is one of the popular real-time models for background subtraction. In this paper, we first extend it from traditional Red-Green-Blue (RGB) color model to multispectral sequences. A self-adaptive mechanism is then designed based on the statistical information extracted from the data themselves, with which the performance has been improved, in addition to saving time and effort to search for the appropriate parameters. Furthermore, the Spectral Information Divergence is introduced to evaluate the spectral distance between the current and reference vectors, together with the Brightness and Spectral Distortion. Experiments on five multispectral sequences with different challenges have shown that the multispectral self-adaptive Codebook model is more capable of detecting moving objects than the corresponding RGB sequences. The proposed research framework opens a door for future works for applying multispectral sequences in moving object detection.

## 1. Introduction

Moving object detection is often the first step in video processing applications, such as transportation, security and video surveillance. A widely used approach for extracting moving objects from the background in the presence of static cameras is detection by background subtraction. Although numerous efforts have been made for this problem and significant improvement has been achieved in recent years, there still exists an insurmountable gap between current machine intelligence and human perception ability. 

### 1.1. Background Subtraction

In the past decade and a half, there have been thousands of researchers devoted to background subtraction and a great number of papers have been published [1]. Although different, most background subtraction techniques share a common denominator: they make the assumption that the observed video sequence is made of a static background, in front of which moving objects, also called foreground, are observed [2]. Thus, background subtraction is sometimes known as foreground detection [3] and foreground–background segmentation [4].

The natural idea of the background subtraction is to automatically generate a binary mask which segments the set of pixels into foreground objects and background. In the ideal case, a simple inter-frame difference between the current frame and a background reference frame is conducted to obtain the mask with the help of a global static threshold. However, detecting moving objects is not as easy as it may first appear, due to the complexity of real-world scenes. Specifically speaking, it is often difficult to obtain a good “empty” background reference frame in the case of a dynamic background, and illumination changes may also make the global static threshold an inferior choice.

During the general background subtraction algorithm, a background model is initially constructed to represent the background information of each pixel, based on a training image sequence via relevant image characteristics, and, subsequently, a distance evaluation between this model and an input image is conducted, resulting in foreground segmentation if the corresponding image features of the input image significantly differ from those of the background model. 

There are several types of background subtraction schemes or machine learning algorithms such as Gaussian mixture models (GMM), Kernel Density Estimation (KDE) and Vibe, to name a few. GMM [5] is one of the most widely used background subtraction methods, where each pixel has been modeled as a mixture of weighted Gaussian distributions. With the probability density function (PDF) learned over a series of training frames, the background subtraction problem becomes a PDF thresholding issue for which a pixel with low probability is likely to correspond to a moving foreground pixel. The main challenge for GMM is that it is a parameter-based algorithm. Thus, its performance is highly influenced by the choice of the selected parameters.

To avoid the difficult question of finding appropriate parameters, nonparametric methods to model background distributions such as KDE have been proposed [6]. Given the previous pixels, the PDF of the intensities in the current frame can be estimated by KDE without any assumption on distribution. However, kernel based methods are computationally intensive.

As a well-known cluster model, the Codebook technique has attracted many researchers’ attention. The motivation for having such a model is that it is fast, efficient, adaptive and able to handle complex backgrounds with sensitivity [7]. In the original Codebook model proposed by [8], a codebook containing several codewords is first constructed from a sequence of RGB sequences from a static camera on a pixel-by-pixel basis; then, the pixel vector of a new frame is compared with the average vector of the tested codeword in the background model, in order to finally obtain a foreground–background segmentation. 

Each method has its advantages over the other and ultimately the type of application and the available data greatly influence the method used for the training of the background subtraction algorithm. Thus, a multitude of more sophisticated methods have been proposed in the recent past. Their efforts mainly focus on two aspects: the first takes on more sophisticated learning modes, while the latter employs more powerful feature representations.

Since the Codebook model is simple and effective, we use it in our background subtraction framework and improve it via modifying the original model and utilizing a new feature-measuring method in the domain of multispectral sequences.

### 1.2. Multispectral Sequences

Earlier studies usually exploit the background subtraction using visible light cameras, mainly RGB, or transferring it to other color model, like YCbCr [9], Lab [3] or YUV (Y, Luminance; U, Chrominance; V, Chroma) color space [10]. Commonly, the methods developed for visible light cameras are particularly sensitive to low light conditions and specular reflections, especially in an outdoor environment. Nowadays, several works propose utilizing alternative kinds of sensors to overcome these limitations. Thanks to the advancements in sensor technologies, now it is possible to capture and visualize a scene at various bands of the electromagnetic spectrum. Thus, one of these alternatives is the use of multispectral cameras which are very robust to bad illumination conditions. 

The corresponding multispectral sequence, as the name implies, is a collection of several monochrome sequences of the same scene and each band, or channel, is taken with additional receptors sensitive to other frequencies of the visible light or to frequencies beyond the visible light like the infrared region of electromagnetic continuum [11]. There is a large potential to improve the detection by multispectral sequences, for the intuitive fact that with more spectral bands, more information could be obtained, particularly for harsh environmental conditions characterized by unfavorable lighting and pronounced shadows, or around-the-clock applications, e.g., surveillance and autonomous driving.

In fact, aforementioned restrictions of visible cameras not only exist in moving object detection, but also in many other vision tasks [12], including (but not limited to) remote sensing [13], food control [14], face recognition [15], semantic segmentation [16], security, defense, space, medical [17], manufacturing and archeology [18]. In this paper, we focus on how to make the most of multispectral sequences for background subtraction framework using the Codebook algorithm. Thus, we propose a Codebook based background subtraction using multispectral sequences. More specifically, we focus our effort on verifying that multispectral sequences do have better performance over traditional RGB sequences. Hereafter, we use the terminology multispectral for any cube that includes more than two spectral bands selected from the original multispectral dataset, to distinguish it from the traditional RGB.

To efficiently achieve this goal, the following incremental contributions have been established. We first extend the original three-dimensional algorithm to the multispectral field. A preliminary version of this step forward appears as part of our previous conference paper [19]. We further present a self-adaptive mechanism of a boundary to get rid of the tiring work of searching for the optimal parameters and combining the spectral information divergence to improve the performance. We have tested this multispectral self-adaptive Codebook model on five multispectral sequences proposed by [20] with different challenges, which show better performance than the corresponding RGB results. This study extends the work in our second conference paper [21].

The rest of this paper is organized as follows: the Codebook algorithm is firstly adapted to multispectral sequences in Section 2. A detailed description of the proposed multispectral self-adaptive mechanism and the utilization of the spectral information divergence are presented in Section 3. Section 4 discusses the experimental evaluation procedure and background subtraction results obtained. Finally, Section 5 summarizes the contributions of this paper and suggests future works.

## 2. Multispectral Codebook 

The Codebook algorithm performs a background subtraction with a clustering technique on sequences taken from a still point of view in order to segment moving objects out of the background. The method works in two phases: Codebook construction and foreground detection. In the first phrase, a model representing the background is constructed from a sequence of images on a pixel-by-pixel basis. Then, in the second phase, every new frame is compared with this background model in order to finally obtain a foreground–background segmentation. 

During the last decade, many works have been dedicated to improving this model. For example, Refs. [22,23] have adopted a two-layer model, to handle dynamic background and illumination variation problems. Other modifications like transferring RGB to other color models in order to solve the problem of existence of shadows and highlights for foreground detection can also be found in [10]. In Ref. [24], a multi-feature Codebook model, which integrates intensity, color and texture information across multiple scales, has been presented.

The object of this work is to investigate the benefits of multispectral sequences rather than traditional RGB to improve the performance of moving objects’ detection. Thus, we first adapt the original Codebook algorithm to multispectral sequences. Minor modifications have been performed comparing with the original RGB Codebook technique [8]. Specifically speaking, the definition of brightness in RGB is extended to multispectral case. In addition, unlike color distortion in the original Codebook, we adopt spectral distortion instead, as the term color is always related to RGB, and, even for three bands out of multispectral sequences, they are not strictly color.

### 2.1. Codebook Construction

For each pixel, a Codebook is constructed to describe what background should act and each codebook $C=\left\{c_{1},\;c_{2},\ldots c_{L}\right\},$ consists of *L* codewords. The number of codewords is different according to the pixel’s activities. More precisely, each codeword is defined by two vectors: the first one contains the average spectral values for each band of the pixel, $v_{m}=\left(V_{1},\;V_{2},\ldots ,V_{n}\right),$ where *n* is the number of bands of multispectral sequences. The second one is a six-tuple vector $aux_{m}={\overset{ˇ}{I}}_{m},\;{\hat{I}}_{m},\;f_{m},\;\lambda_{m},\;p_{m},\;q_{m}$, where:

- ${\overset{ˇ}{I}}_{m},\;{\hat{I}}_{m}$, the min and max brightness, respectively, of all pixels assigned to codeword $c_{m}$.
- $f_{m}$, the frequency with which codeword $c_{m}$ has occurred.
- $\lambda_{m}$, the maximum negative run-length (MNRL), defined as the longest interval of time during the construction period that the codeword $c_{m}$ has not been updated.
  $p_{m},\;q_{m}$, the first and the last times, respectively, that the codeword $c_{m}$ has been occurred.

To construct this background model, the codebook for each pixel is initialized as the first line in Algorithm 1 shows, when the algorithm starts. N is defined as the total number of frames in the construction phase. Then, the current value $x_{t}$ of a given pixel is compared to its current codebook. If there is a match with a codeword $c_{m},$ this codeword is used as the sample’s encoding approximation. Otherwise, a new codeword is to be created. The detailed algorithm of Codebook construction is given in Algorithm 1, during which the matching process is evaluated by two judging criteria: (a) brightness bounds and (b) spectral distortion.
**Algorithm 1** Codebook Construction$L⟵0^{1},\;C⟵\phi$$\mathrm{for}\;t=1⟶N\;\mathrm{do}\;x_{t}=\left(X_{1},\;X_{2},\ldots ,X_{n}\right),\;\;I=\sqrt{\sum_{i=1}^{n}X_{i}^{2}}$find the matching codeword to ***x_t_*** in **C** if (a) and (b) occur.***(a) brightness***$\left(I,\;\langle {\overset{ˇ}{I}}_{m},\;{\hat{I}}_{m}\rangle \right)$ = ***true******(b) spectral**_**dist***$(x_{t},\;v_{m})\leq \varepsilon_{1}$if ***C***⟵***ϕ*** or there is no match, then ***L***⟵***L*** + **1**, create a new codeword***v*_0_** = ***x_t_******aux*_0_** = 〈***I***,***I***,**1**,***t***−**1**,***t***,***t***〉.Else, update the matched codeword, composed of$v_{m}=\left({\bar{X}}_{m1},\;{\bar{X}}_{m2},\;\ldots \;{\bar{X}}_{mn}\right)\;\mathrm{and}\;aux_{m}=\;\langle {\overset{ˇ}{I}}_{m},\;{\hat{I}}_{m},\;f_{m},\;\lambda_{m},\;p_{m},\;q_{m}\rangle \;\mathrm{by}\;\mathrm{editing}$$v_{m}\;\leftarrow \;\left(\frac{f_{m}{\bar{X}}_{m1}+X_{1}}{f_{m}+1},\;\frac{f_{m}{\bar{X}}_{m2}+X_{2}}{f_{m}+1},\ldots ,\frac{f_{m}{\bar{X}}_{mn}+X_{n}}{f_{m}+1}\right)$$aux_{m}\leftarrow \langle min\left\{I,\;{\overset{ˇ}{I}}_{m}\right\},\;max\left\{I,\;{\hat{I}}_{m}\right\},\;f_{m}+1,\;max\left\{\lambda_{m},t-q_{m}\right\},\;p_{m},\;t\rangle$end for

(a) The brightness of the pixel must lie in the interval $\left[I_{low},I_{hi}\right]$. For grayscale pixels, the grayscale value or the brightness is obtained by $I=\left|x\right|=\sqrt{x^{2}}$. For RGB pixels, the brightness is calculated by $I=\sqrt{R^{2}+G^{2}+B^{2}}$. Accordingly, for multispectral pixel vector $x_{t}=\left(X_{1},\;X_{2},\ldots ,X_{n}\right),$ the brightness can be also measured by the L2-norm of the pixel vector

(1) $$I=\sqrt{\sum_{i=1}^{n}x_{i}^{2}},$$

where *n* is the number of bands. The boundaries are calculated from the min and max brightness ${\overset{ˇ}{I}}_{m},\;{\hat{I}}_{m}$, with Equation (2):

(2) $$I_{low}=\alpha {\hat{I}}_{m},I_{hi}=min\;\left\{\beta {\hat{I}}_{m}\;,\frac{{\overset{ˇ}{I}}_{m}}{\;\alpha }\right\},$$

where the values of α and β are obtained from experiments. Typically, α is between 0.4 and 0.7, and β is between 1.1 and 1.5 [8].

Thus, the logical brightness function is defined as:

(3) $$brightness\left(I,\;\langle {\overset{ˇ}{I}}_{m},\;{\hat{I}}_{m}\rangle \right)=\{\begin{array}{l} true\;if\;I_{low}\leq I\leq I_{hi},\\ false\;otherwise. \end{array}$$

(b) The spectral distortion, spectral_dist, must lie under a given threshold *ε_1_*. Following Equations (4) and (5), define the calculation of spectral distortion between an input multispectral vector $x_{t}=\left(X_{1},\;X_{2},\ldots ,X_{n}\right)$ and a background average multispectral vector $v_{m}=\left(V_{1},\;V_{2},\ldots ,V_{n}\right)$:

(4) $$p^{2}=\;∥x_{t}{∥}^{2}cos^{2}\theta =\frac{x_{t},\;v_{m}{}^{2}}{∥v_{m}{∥}^{2}},$$

(5) $$spectral\_dist\left(x_{t},\;v_{m}\right)=\sqrt{∥x_{t}{∥}^{2}-p^{2}}.$$

**Figure 1:**
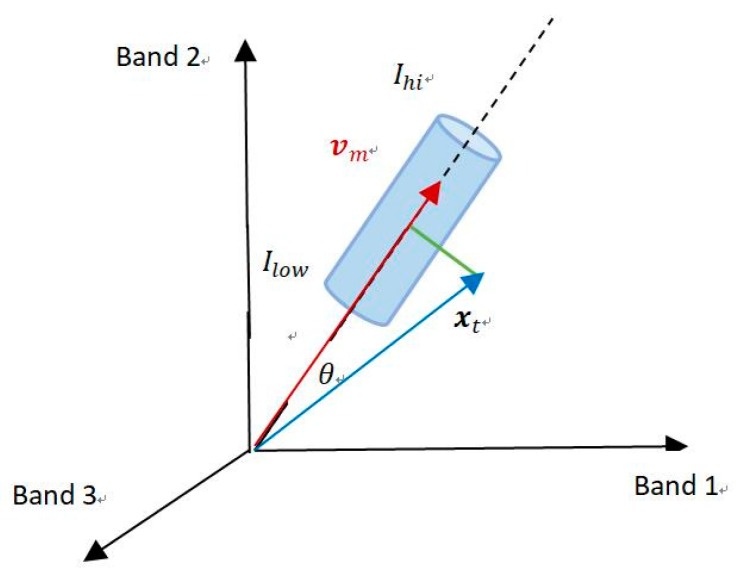
Visualization of the judging criteria (a) brightness bounds and (b) spectral distortion.

**Figure 2:**
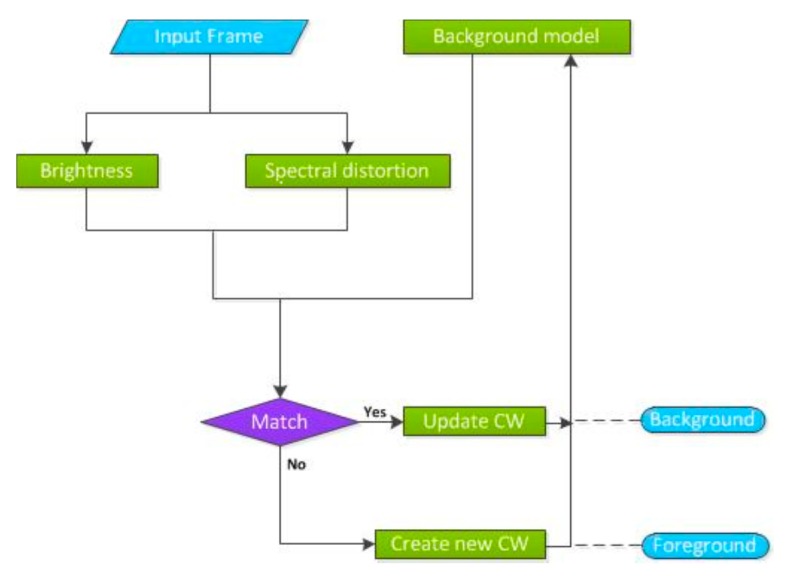
Codebook algorithm.

To make it intuitive, the two criteria (a) and (b) are visualized in Figure 1. The pixel of a multispectral image is considered as a vector in an *n*-dimensional space and three bands are used as an example. In Figure 2, the blue cylinder represents a certain codeword, whose bottom radius is the spectral distortion threshold $\varepsilon_{1}$. The red and the blue vectors stand for the average spectral $v_{m}$ in this codeword and the current pixel $x_{t}$, respectively. With Equations (4) and (5), the spectral distortion can be calculated and illustrated with the green line. As discussed above, a match is found if the brightness of the pixel vector lies between $I_{\mathrm{low}}$ and $I_{\mathrm{hi}}$, and the spectral distortion is under a given threshold $\varepsilon_{1}$. Accordingly, the L2-norm of vector $x_{t}$ must be located along the axis in the cylinder and the length of the green line must be smaller than the radius of the cylinder.

At the end of the Codebook construction algorithm, the model has to clean the codewords that are most probably belonging to foreground objects. To achieve that, the algorithm makes use of MNRL recorded in the six-tuple of each codeword. A low value means that the codeword has been frequently observed. A high value means that it has been less frequently observed and that it should be removed from the model as it is probably part of foreground. The threshold value is often set as half of the number of images used in the construction period [8].

### 2.2. Foreground Detection

The foreground detection phase that follows performs almost the same task as that of the construction phase. It simply consists in testing the difference of the current image from the background model with respect to brightness and spectral distortion. The pixel is detected as foreground if no acceptable matching codeword exists. Otherwise, it is classified as background and the corresponding codeword is updated at the same time. During the detection phase, the threshold for spectral distortion is set with higher value to be more tolerant for noise. 

The process of the Codebook technique is illustrated in Figure 2, where the dashed lines represent the decision made in the detection phase. CW is codeword for short. We need to note that, in the construction phase, when there is no appropriate match found, a new codeword will be established, while, in the detection phase, the pixel is detected as foreground directly and no more extra measures will be taken.

## 3. Multispectral Self-Adaptive Codebook 

In this section, we proposed two techniques to improve the Codebook algorithm: multispectral self-adaptive mechanism and new estimation criteria. With the first technique, the brightness bound and spectral distortion thresholds are calculated automatically from the image data themselves statistically, not chosen empirically like the original Codebook, which is helpful for researchers to get rid of the cumbersome task of parameters tuning. Furthermore, the spectral information divergence is employed to be the criteria to evaluate the distance in the matching process.

### 3.1. Self-Adaptive Mechanism 

Like other parametric methods, the detection result of the original Codebook is heavily impacted by the parameters. The Codebook model devoted to in Section 2 has the four following basic key parameters: α, β, $\varepsilon_{1}$ and $\varepsilon_{2}$. To be specific, α and β are used to obtain the bounds from the min and max brightness ${\overset{ˇ}{I}}_{m}$ and ${\hat{I}}_{m}$ in a certain codeword, with Equation (2), and, $\varepsilon_{1}$ and $\varepsilon_{2}$ are the spectral distortion thresholds used in the construction and detection phases, respectively. 

The fashionable way to get these parameters is empirical and experimental. The pioneers of this technique [8] have provided the typical range of these parameters. However, this is still far from adequate because manual parameter tuning is still required to achieve satisfying results for a specific scene, which is always a really cumbersome and tricky task for researchers. In addition, if the algorithm needs to be run for long periods of time, the parameters should be automatically adjusted according to the environmental changes. What is more important for our research objective, when using the multispectral sequences, is that the parameters should change with different numbers of bands. Therefore, there is a need for further research with regard to realizing an automatic selection for optimal parameters.

Motivated by the work of [9], which has proposed the statistical parameter estimation method in YCbCr color space, we propose a multispectral self-adaptive method for automatically optimal parameter selection. That is to say, those parameters do not need to be obtained by burdensome experiments, but to be estimated from the data themselves statistically, which can help to save a lot of efforts and time.

Firstly, the statistical information is calculated iteratively and recorded for each codeword during the process of constructing the background model. In spite of the vector of average spectral values of the pixel $v_{m}$ and the six-tuple $aux_{m}$, we record another vector named $S_{m}$, which represents the set of the variance of the separate spectrum $\sigma_{i}^{2}$. At the same time, for $v_{m}$ and $S_{m}$, one more dimension is added to record the average ${\hat{I}}_{m}$ and the variance $\sigma_{I}^{2}$ of brightness $\sigma_{I}$. Thus, for the *n*-channel combination out of multispectral sequences, the $v_{m}$ and $S_{m}$ vectors are of *n*+*1* dimension. The extra one channel stands for the numerical information of the brightness. Referring to the algorithm illustrated in Algorithm 1, the initialization and update strategy are kept the same for the six-tuple vector $aux_{m}$, while they are modified a little for $v_{m}$. Specifically speaking, for a new codeword of a given pixel, the initialization of $v_{m}$ is:

(6) $$v_{0}=\left(X_{1},\;X_{2},\ldots ,X_{n}\;,\;I\right)\;,$$

and when there is a match with this certain codeword, $v_{m}$ is updated as below:

(7) $$v_{m}\;\leftarrow \;\left(\frac{f_{m}{\bar{X}}_{m1}+X_{1}}{f_{m}+1},\;\frac{f_{m}{\bar{X}}_{m2}+X_{2}}{f_{m}+1},\ldots ,\frac{f_{m}{\bar{X}}_{mn}+X_{n}}{f_{m}+1},\frac{f_{m}{\bar{I}}_{m}+I_{n}}{f_{m}+1}\right).$$

Meanwhile, the $S_{m}$ of the new codeword is initialed with

(8) $$S_{0}=\left(X_{1}^{2},\;X_{2}^{2},\ldots ,\;X_{n}^{2},\;I^{2}\right)$$

and updated with

(9) $$\begin{array}{l} S_{m}\\ \;\leftarrow \;\left(\frac{f_{m}{\bar{\sigma }}_{m1}^{2}+{\left(X_{1}-{\bar{X}}_{m1}\right)}^{2}}{f_{m}+1},\;\frac{f_{m}{\bar{\sigma }}_{m2}^{2}+{\left(X_{2}-{\bar{X}}_{m2}\right)}^{2}}{f_{m}+1},\ldots ,\frac{f_{m}{\bar{\sigma }}_{nm}^{2}+{\left(X_{n}-{\bar{X}}_{mn}\right)}^{2}}{f_{m}+1},\frac{f_{m}{\bar{\sigma }}_{mI}^{2}+{\left(I-{\bar{I}}_{m}\right)}^{2}}{f_{m}+1}\right) \end{array}$$

Then, these statistics are used to build the self-adaptive Codebook algorithm, where the definitions of brightness and spectral distortion are kept the same with those in the last section, as shown in Equations (1), (4) and (5). During the matching process, the statistical information calculated and recorded above for each codeword is used to estimate both the brightness bounds and spectral distortion threshold. To be specific, the bounds of brightness can be estimated by

(10) $$I_{low}={\overset{ˇ}{I}}_{m}-\sigma_{I},\;I_{hi}={\hat{I}}_{m}+\sigma_{I},$$

where $\sigma_{I}$ is the standard deviation of brightness in the current codeword, whose square is the last element of $S_{m}$. In addition, the threshold ε for the spectral distortion for both phases is calculated by

(11) $$\varepsilon =\max\;\left(\left[\sigma_{1\;}\;\sigma_{2}\ldots \ldots \;\sigma_{n\;}\right]\right),$$

where is the standard deviation of the *i*th band value in the current codeword, whose square is the corresponding *i* th element of $S_{m}$.

With the self-adaptive mechanism, brightness bounds and spectral distortion threshold are able to adjust themselves with statistical properties of the input sequences. In the phase of background construction, for each pixel, when a new image arrives, the brightness and spectral distortion are first computed using Equations (1), (4) and (5); then, the matching process is conducted codeword by codeword. If (a) the brightness of the new pixel lies in the current interval of the brightness bounds and (b) the spectral distortion is smaller than the current threshold of a certain codeword, the new pixel will be modeled as a perturbation on this background codeword, whose brightness bounds and the spectral distortion threshold will subsequently be updated using Equations (10) and (11). Unless, a new codeword will be seeded. Later on, the similar task is performed in the detection phase. The new pixel is classified as background if an acceptable matching codework exists and the codeword will be also updated using Equations (10) and (11) at the same time. Otherwise, the pixel is detected as foreground.

### 3.2. Spectral Information Divergence

As illustrated above, the main idea of Codebook background model construction is that, if the pixel vector of the current image is close enough to the average vector of the tested codeword in the background model, it will be regarded as a perturbation on that codeword, unless it will establish a new codeword to be associated with that pixel. However, how can this closeness, or in another way of comprehension, distance be measured? 

In the aforementioned Codebook algorithm, two criteria have been adopted to evaluate the distance between two vectors, the brightness (B) and the spectral distortion (SD). Specifically speaking, the brightness is simply the L2-norm of the related bands, and the spectral distortion is measured as a function of the brightness-weighted angle between the current and reference spectral vectors, as illustrated in Equations (4) and (5). We should be aware that the brightness and spectral distortion defined previously are only one way of estimation criteria.

Here, we adopt another information-theoretic spectral measure, referred to as Spectral Information Divergence (SID) [25], which is applied to determine the spectral closeness or distance between two multispectral vectors. SID models the spectral band-to-band variability as a result of uncertainty caused by randomness, which is based on the Kullback–Leibler divergence to measure the discrepancy of probabilistic behaviors. That is to say, it considers each pixel as a random variable and then defines the desired probability distribution by normalizing its spectral histogram to unity, which is expressed by Equation (12)

(12) $$P_{x}\left(i\right)=\frac{x_{t}\left(i\right)}{\sum_{i=1}^{n}x_{t}\left(i\right)}\;P_{v}\left(i\right)=\frac{v_{m}\left(i\right)}{\sum_{i=1}^{n}v_{m}\left(i\right)},$$

where *n* is the number of bands. Then, the spectral information divergence $d_{SID}$ between the current spectral vector $x_{t}$ and the background model $v_{m}$ can now be defined with Equation (13)

(13) $$d_{SID}\left(x_{t},\;v_{m}\right)=\;\overset{n}{\underset{i=1}{\sum }}P_{x}\left(i\right)log\frac{P_{x}\left(i\right)}{P_{v}\left(i\right)}+\overset{n}{\underset{i=1}{\sum }}P_{v}\left(i\right)log\frac{P_{v}\left(i\right)}{P_{x}\left(i\right)}.$$

If the spectral information divergence is employed to replace the spectral distortion in the previous Codebook model to be the judging criteria together with the brightness condition, the brightness and spectral information divergence are first computed when a new frame arrives and the main construction and detection procedures are similar. The threshold updating strategy for spectral information divergence is kept the same with that for spectral distortion. That is to say, in the matching process, we do not need to search the parameters. To further utilize the information, the three criteria mentioned in this paper can be employed together. This step forward opens a door for other possibilities to seek a novel kind of feature-measuring methods in the construction of the Codebook background model.

## 4. Experiments

### 4.1. Dataset

To evaluate the performance of the proposed approach, a multispectral dataset [20], which is composed of five challenging video sequences containing between 250 and 2300 frames of size 658 × 491, is adopted for testing. This is the first multispectral dataset available for research community in background subtraction. These sequences are all publicly available, and the ground truth sequences are already obtained by manual segmentation.

**Figure 3:**
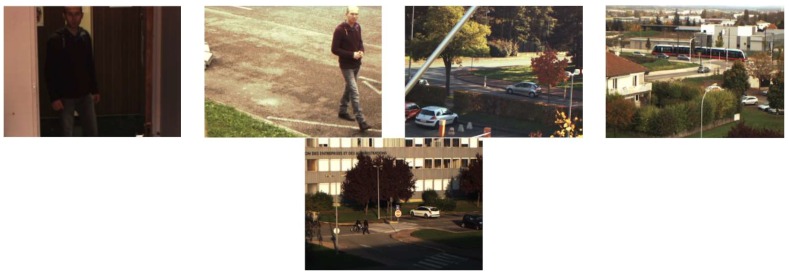
Examples of the dataset [20].

The acquisition of this dataset is performed with a commercial camera, the FD-1665-MS, from FluxData, Inc. (Rochester, MN, USA). It can acquire seven spectral narrow bands simultaneously, six in the visible spectrum and one in the near infrared. In addition, the RGB sequences can be easily obtained with a linear integration of the original multispectral sequences weighted by three different spectral envelopes. Therefore, each scene consists of a multispectral sequence of size 658 × 491 × 7 for each frame and the corresponding RGB sequence of size 658 × 491 × 3. Figure 3 presents examples of RGB sequences of the five scenes. Note that the first scene is indoors, while the other four are outdoors with different challenges such as tree shadows, faraway intermittent objects and objects with shadows. 

### 4.2. Experiment Results

#### 4.2.1. Multispectral Codebook 

Since the traditional Codebook algorithm for RGB, is three-bands-based, we begin with the trials with three bands. The number of combination composed of three bands out of seven is $c_{7}^{3}$ = 35. For fair comparison, parameters for RGB and these three-dimensional multispectral sequences are the same for the experiments. The four parameters, which are empiric values determined experimentally and used for the Codebook algorithms are as the following:

$$\alpha =0.7\;\beta =1.5\;\varepsilon_{1}=0.02\;\varepsilon_{2}=0.04.$$

**Figure 4:**
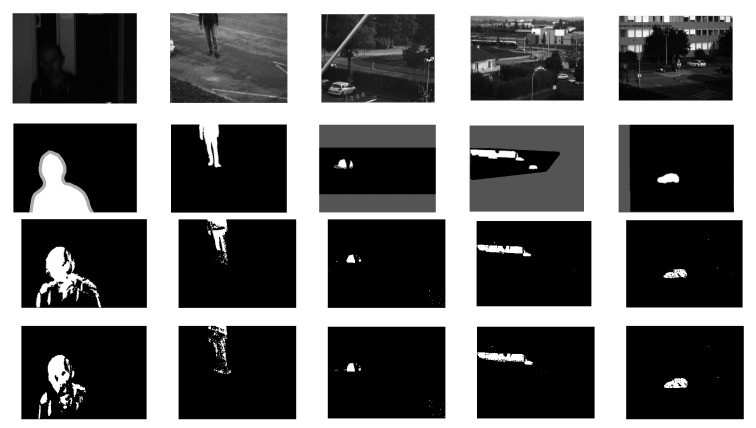
Background subtraction results on five videos. The top row is original multispectral sequences. The second row is the corresponding ground truth. The third and fourth are the results obtained by the respective best combination of multispectral and RGB sequences.

The tests are conducted on the five different video sequences. For evaluation, the well-known F-measure is computed for each combination of each video sequence with its available ground truth data and illustrated in Table 1. The RGB results are also shown in the last row, acting as a reference. The largest value in each column is in bold. Some visual examples are shown in Figure 4. For all sequences, no morphological operation is applied.

Table 1 shows the performance comparison between the three-dimensional multispectral sequences and RGB, whose result is not the best for all five videos. The average of the F-Measure on the five videos is calculated and listed in the farthest right column, from which the results (0.8399) of the best average three-band combination on five videos present nearly 3% improvement than the result (0.8113) of RGB. As it is shown, the multispectral sequences can represent an alternative to conventional RGB sequences in the background subtraction.

#### 4.2.2. Multispectral Self-Adaptive Codebook

In this part, the self-adaptive mechanism and spectral information divergence illustrated in Section 3 have been adopted for *n* bands from five multispectral sequences.

Firstly, the experiments are conducted on the thirty-five different three-band-based combinations, thirty-five different four-band-based combinations, twenty-one different five-band-based combinations, seven different six-band-based combinations, and total seven-band, together with the RGB for five videos. Then, the largest F-measures are selected and listed in Table 2. As a reminder, Brightness (B) and Spectral Distortion (SD) are used for evaluating the distance between two pixel vectors, like what are used in 4.2.1. In Table 2, the largest F-measure for each video is in bold and the average F-measures for *n* bands of multispectral sequences on five videos are listed in the last row. From Table 2, multispectral sequences always outperform the corresponding RGB sequences, among which the seven-band-based combination performs worst, but still can be nearly 3% more than the RGB result.

In the following experiment, the spectral information divergence explained in Section 3.2 is used to replace the spectral distortion in the matching process. The experiments for different *n*-band-based multispectral sequences are conducted and the largest F-measures are selected and listed in Table 3. Here, the judging criteria are brightness (B) and Spectral Information Divergence (SID). With this new set of criteria, the multispectral sequences still have better performance than the RGB sequences. The same as in Table 2, the four-band-based combination achieves the best results. 

In the last experiment, brightness (B), spectral distortion (SD) and spectral information divergence (SID) are adopted together to determine the distance between two spectral vectors, during which the self-adaptive threshold is shared by spectral distortion and spectral information divergence. From the results from the five videos and each combination, the best F-measures for each column are extracted and listed in Table 4.

From Table 2, Table 3 and Table 4, regardless of different judging criteria used in the matching process, multispectral sequences show an attractively better performance than the traditional RGB sequences. Then, the best multispectral results from Table 1, Table 2, Table 3 and Table 4 are summarized in Table 5, together with the corresponding RGB results.

In Table 5, the first category, using brightness and spectral distortion with a static parameter mechanism, records the best multispectral and RGB results of each sequence taken from Table 1. In the self-adaptive mechanism, the same items for three different sets of criteria are also extracted from Table 2 to Table 4. The corresponding average F-measures on the five sequences are calculated and listed in the last column. 

From Table 5, we can see that, on the Videos 2 to 5, which are outdoor scenes, it performs best to adopt the multispectral self-adaptive technique using the brightness (B) and spectral distortion (SD) as matching criteria. What needs to be mentioned is that, in this process, researchers do not have to take time and effort to search for the appropriate parameters. For the indoor Video 1, the utilization of the spectral information divergence (SID) does great help. The F-measure shows a great jump when SD is replaced by SID. When the three criteria are used together, the performance drops a little from the B+SID combination but is still far better than that of B+SD. If all videos are considered, judging from the mean F-measures, the three-criteria-based multispectral self-adaptive Codebook is the most promising choice. 

## 5. Conclusions and Perspectives

In this paper, we have proposed a new framework for background subtraction by investigating the advantages of multispectral sequences with the Codebook model. Given the pioneering work of Codebook algorithm, we have achieved significant improvements. Firstly, the original Codebook algorithm is adapted to multispectral sequences. Furthermore, a self-adaptive mechanism is designed to obtain the parameters based on the statistical information extracted from the data themselves. The parameters in the original version are selected empirically and experimentally. This makes the algorithm not solid, reliable and robust, as the detection results of Codebook are heavily impacted by the parameters, let alone the time and effort to search for the optimal parameters. Furthermore, the spectral information divergence is then introduced in the matching process to further improve the performance. The results clearly show that the multispectral self-adaptive Codebook is more capable of detecting moving objects and it is very convenient to be applied in other multispectral datasets with different numbers of bands. This research framework forward opens a door for future works for applying multispectral sequences for robust detection and motion analysis of moving targets. One future work is to explore other powerful feature representations extracted from multispectral sequences, like texture, to further improve the accuracy of background subtraction. Another potential direction is to measure the degree of stability via the intensity of the distribution of all the multispectral bands and select the most stable bands for background subtraction to make better use of multispectral sequences. In addition, we would like to further investigate the multispectral dataset and improve our current work comparing with other prior works.

## Figures and Tables

**Table 1:** Average F-Measures on the five videos (3-band case).

	Combination	Video 1	Video 2	Video 3	Video 4	Video 5	Mean
1	123	0.6505	0.9422	0.7733	0.8037	0.7211	0.7782
2	124	0.8355	0.9420	0.7516	0.8065	0.7864	0.8244
3	125	0.8342	0.9450	0.7515	0.8148	0.7734	0.8238
4	126	0.7104	0.8950	0.7082	0.8047	0.8154	0.7867
5	127	0.7739	0.9396	0.6558	0.8071	0.7247	0.7802
6	134	0.8421	0.9461	**0.7921**	0.8513	0.7670	**0.8397**
7	135	0.8402	0.9538	0.7838	0.8350	0.7622	0.8350
8	136	0.7017	0.9040	0.7417	0.8381	0.8031	0.7977
9	137	0.7764	0.9463	0.6908	0.8354	0.7113	0.7920
10	145	0.8636	0.9440	0.7689	0.8475	0.7757	**0.8399**
11	146	0.8519	0.8952	0.7296	0.8435	0.8132	0.8267
12	147	0.7932	0.9488	0.6881	0.8084	0.7480	0.7973
13	156	0.8705	0.9038	0.7564	0.8440	0.8091	0.8368
14	157	0.7943	0.9538	0.6908	0.8252	0.7361	0.8000
15	167	0.7839	0.9302	0.6518	0.8105	0.7832	0.7919
16	234	0.8358	0.9434	0.7418	0.8168	0.7810	0.8238
17	235	0.8339	0.9448	0.7345	0.8180	0.7657	0.8194
18	236	0.6959	0.8849	0.6880	0.8104	0.8075	0.7773
19	237	0.7714	0.9459	0.6400	0.8259	0.7248	0.7816
20	245	0.8583	0.9386	0.7099	0.8199	0.7899	0.8233
21	246	0.8437	0.8795	0.6677	0.7901	**0.8241**	0.8010
22	247	0.7900	0.9467	0.6273	0.8083	0.7662	0.7877
23	256	0.8666	0.8831	0.6832	0.8000	0.8179	0.8102
24	257	0.7893	0.9472	0.6262	0.8211	0.7539	0.7875
25	267	0.7838	0.9050	0.5934	0.8221	0.7923	0.7793
26	345	0.8619	0.9423	0.7409	0.8585	0.7746	0.8356
27	346	0.8436	0.8831	0.7088	0.8423	0.8076	0.8171
28	347	0.7904	0.9455	0.6525	0.8116	0.7634	0.7927
29	356	0.8661	0.8904	0.7187	**0.8650**	0.8026	0.8286
30	357	0.7894	**0.9546**	0.6640	0.8298	0.7577	0.7991
31	367	0.7833	0.9169	0.6281	0.8308	0.7773	0.7873
32	456	**0.8718**	0.8799	0.6992	0.8297	0.8131	0.8187
33	457	0.7897	0.9402	0.6435	0.8140	0.7690	0.7913
34	467	0.7854	0.9060	0.6181	0.8071	0.7904	0.7814
35	567	0.7844	0.9098	0.6095	0.8027	0.7869	0.7787
36	RGB	0.8086	0.9431	0.7578	0.7679	0.7789	0.8113

**Table 2:** Best F-measures with B+SD on five videos.

B+SD	3 Bands	4 Bands	5 Bands	6 Bands	7 Bands	RGB
Video 1	0.7995	0.8046	**0.8060**	0.8043	0.7983	0.4789
Video 2	0.9615	0.9624	0.9636	**0.9643**	0.9631	0.9535
Video 3	0.9231	**0.9248**	0.9204	0.9051	0.8381	0.9188
Video 4	0.8981	**0.9001**	0.8999	0.8918	0.8856	0.8871
Video 5	0.9171	**0.9198**	0.9190	0.9189	0.9110	0.9130
mean	0.8999	0.9023	0.9018	0.8969	0.8792	0.8303

**Table 3:** Best F-measures with B+SID on five videos.

B+SID	3 Bands	4 Bands	5 Bands	6 Bands	7 Bands	RGB
Video 1	0.9208	**0.9219**	0.9059	0.8676	0.7883	0.6355
Video 2	**0.9538**	0.9526	0.9504	0.9471	0.9451	0.9479
Video 3	**0.8939**	0.8914	0.8825	0.8766	0.8351	0.8867
Video 4	0.8784	**0.8807**	0.8783	0.8728	0.8558	0.8217
Video 5	0.8765	0.8801	**0.8842**	0.8425	0.7855	0.8447
mean	0.9047	0.9053	0.9003	0.8813	0.8420	0.8273

**Table 4:** Best F-measures with B+SD+SID on five videos.

B+SD+SID	3B	4B	5B	6B	7B	RGB
Video 1	0.9144	**0.9147**	0.8971	0.8607	0.7727	0.6555
Video 2	0.9614	0.9623	0.9635	**0.9642**	0.9631	0.9535
Video 3	**0.9213**	0.9180	0.8938	0.8634	0.8045	0.9054
Video 4	0.8968	**0.8979**	0.8972	0.8885	0.8821	0.8867
Video 5	0.8791	0.8800	**0.8948**	0.8459	0.7853	0.8543
mean	0.9146	0.9146	0.9093	0.8845	0.8415	0.8511

**Table 5:** Best F-measures with different mechanisms and sets of criteria on the five videos.

Mechanism	Criteria	Sequences	Video 1	Video 2	Video 3	Video 4	Video 5	Mean
Static parameters	B+SD	RGB	0.8086	0.9431	0.7578	0.7679	0.7789	0.8113
		Multi	0.8718	0.9546	0.7921	0.8650	0.8241	0.8615
Self-adaptive mechanism	B+SD	RGB	0.4789	0.9535	0.9188	0.8871	0.9130	0.8303
		Multi	0.8060	0.9643	0.9248	0.9001	0.9198	0.9030
	B+ SID	RGB	0.6355	0.9479	0.8867	0.8217	0.8447	0.8273
		Multi	0.9219	0.9538	0.8939	0.8807	0.8842	0.9069
	B+SD+SID	RGB	0.6555	0.9535	0.9054	0.8867	0.8543	0.8511
		Multi	0.9147	0.9642	0.9213	0.8979	0.8948	0.9186
